# Palladium Decorated *N*-Doped Carbon Foam as a Highly Active and Selective Catalyst for Nitrobenzene Hydrogenation

**DOI:** 10.3390/ijms23126423

**Published:** 2022-06-08

**Authors:** Ádám Prekob, Ákos Szamosvölgyi, Gábor Muránszky, János Lakatos, Zoltán Kónya, Béla Fiser, Béla Viskolcz, László Vanyorek

**Affiliations:** 1 Institute of Chemistry, University of Miskolc, 3515 Miskolc, Hungary; kempadam@uni-miskolc.hu (Á.P.); kemmug@uni-miskolc.hu (G.M.); mtasotak@uni-miskolc.hu (J.L.); bela.viskolcz@uni-miskolc.hu (B.V.); 2 Department of Applied and Environmental Chemistry, University of Szeged, Rerrich Béla tér 1, 6720 Szeged, Hungary; akos.szamosvolgyi@gmail.com (Á.S.); konya@chem.u-szeged.hu (Z.K.); 3 Higher Education and Industrial Cooperation Centre, University of Miskolc, 3515 Miskolc, Hungary; 4 Transcarpathian Hungarian College of Higher Education, 90200 Beregszász, Transcarpathia, Ukraine

**Keywords:** carbon foils, aniline, carbon snake, catalytic hydrogenation, catalyst support

## Abstract

Carbon foam was synthesized by the carbonization of 4-nitroaniline. The reaction is an alternative of the well-known “carbon snake” (or sugar snake) demonstration experiment, which leads to the formation of nitrogen-doped carbon foils due to its nitrogen content. The synthesized carbon foils were grinded to achieve an efficient catalyst support. Palladium nanoparticles were deposited onto the surface of the support, which showed continuous distribution. The prepared Pd nanoparticle decorated carbon foils showed high catalytic activity in nitrobenzene hydrogenation. By applying the designed catalyst, total nitrobenzene conversion, a 99.1 n/n% aniline yield, and an exceptionally high selectivity (99.8 n/n%) were reached. Furthermore, the catalyst remained active during the reuse tests (four cycles) even without regeneration.

## 1. Introduction

Catalytic hydrogenation is a widely used process in the pharmaceutical and chemical industries [1]. More than 90% of the chemical production processes include at least one catalytic step, usually a hydrogenation reaction. The hydrogenation of nitro compounds is the most often used technique to prepare amino functional groups in a molecule [2]. Various catalysts, such as carbon- or metal oxide-based noble metals or transitional metals, can be used for this purpose [3,4,5]. However, Pd seems to be the most efficient catalyst in various reactions used in fine chemical synthesis such as C-C coupling, hydrogenolysis, hydrogenation, oxidation, and dehydrogenation [6,7,8,9,10]. The catalytic hydrogenation of nitrobenzene is an important industrial process as part of polyurethane, drug, and dye production; furthermore, it can be used as a model reaction for nitro compounds. Other than the optimal reaction conditions, the activity and selectivity of the applied catalysts are the most important factors to achieve a highly efficient industrial production process. Since Pd is a perfect choice for hydrogenation reactions, it is commonly used in combination with different supports [11,12,13,14,15]. Metal oxide supported catalysts seem to be a suitable choice due to their easy handling and separation from the reaction media, but their application causes the formation of higher amounts of azo-, azoxy-, and other derivatives to the detriment of the main product (aniline) [16,17]. On the other hand, catalysts with various carbon-based supports such as activated carbon, carbon nanotubes, and fullerene provide higher aniline selectivity and, due to their large specific surface area, a higher reaction rate, and thus enhanced aniline production can be achieved [18,19,20].

Carbon-based supports are mainly prepared by the carbonization of different carbon-containing materials such as coconut shells, corn cobs, or even waste tires [21,22,23,24,25]. The produced carbon-based materials are excellent heat and electric conductors, with fine mechanical resistance and high specific surface areas. To further improve the advantageous properties of a carbon-based material, doping is often applied, which has led to modified geometrical and electronic structures [26,27,28]. By decomposing nitrogen-containing carbon sources (e.g., amines), *N*-doped carbon structures can be prepared [29,30]. *N*-doped carbon structures provide beneficial properties for the catalysts by creating the appropriate circumstances for the growth of the catalytic metal particles, thereby reducing their size, which makes the catalysts more efficient [30,31,32,33,34].

A “carbon snake” is a foam-like carbon structure which is prepared by the reaction of 4-nitroaniline (or sugar) and concentrated sulfuric acid at an elevated temperature [35]. Foam structured carbon materials have a high specific surface area and easily adjustable thermal, electrical, and surface properties [36]. Due to their advantageous characteristics, they can be used as adsorbents, electrode materials, or catalyst supports [36,37,38,39,40] The effective adsorption and surface properties can be well utilized for catalytic purposes. Using 4-nitroaniline as a nitrogen-containing carbon source, a nitrogen-doped “carbon snake” can be prepared. The reaction is a popular scientific communication and demonstration experiment for dehydration called sugar snake; similar to the Pharaoh’s snake or elephant toothpaste reactions, it provides a foam-like product [41,42]. It has a very high reaction speed and as a result provides an extremely light foam with a special structure. The “carbon snake” has already been tested as an electrocatalyst material for Zn-air batteries and provided better results than commercial catalysts [43].

As a potential catalyst support material, a “carbon snake” was synthesized and applied to prepare carbon foil (CF) which was used as a catalyst support and decorated with Pd nanoparticles. The final Pd/CF catalyst was characterized and tested in nitrobenzene hydrogenation.

## 2. Materials and Methods

### 2.1. Materials

For the preparation of the carbon-based foam, 4-nitroaniline (Alfa Aesar GmbH, 76185 Karlsruhe, Germany) and sulfuric acid (95 wt%, VWR Intern. S.A.S, F-94126 Fontanay-sous-Bois, France) were used. Carbon dioxide was used to activate the prepared foam (Gourmet, Messer). Palladium(II) nitrate dihydrate (Pd(NO_3_)_2_*2H_2_O, Alfa Aesar Ltd., Ward Hill, 01835 MA, USA) was applied to deposit Pd onto the carbon foil support. Nitrogen (purity 4.0, Messer) and hydrogen (purity 4.0, Messer) were used during the experiments. Nitrobenzene (NB, Acros Organics, Fair Lawn, 07950 NJ, USA) was used as reactant during the catalytic hydrogenation tests. The applied analytical standards (azobenzene, nitrosobenzene, *N*-methylaniline) were purchased from Sigma-Aldrich Co. (St. Louis, 63118 MO, USA).

### 2.2. Characterization Techniques

The structure of the carbon foam was studied by high-resolution scanning electron microscope (SEM) applying a Helios G4 PFIB CXe (Thermo Scientific, Waltham, MA, USA) instrument and using carbon tape for sample preparation. The morphology and particle size of the prepared materials were examined by high-resolution transmission electron microscopy (HRTEM, FEI Technai G2 electron microscope, 200 kV, Rodovia Washington Luis, Brazil). The samples were prepared by dropping the aqueous suspension of the nanoparticles on 300 mesh copper grids (Ted Pella Inc., Redding, CA, USA). The zeta potential of the grinded carbon foam was measured in the aqueous phase by determining the electrophoretic mobility of the particles (laser Doppler electrophoresis) using a Malvern Zetasizer Nano ZS. The functional groups on the surface of the samples were identified by using a Bruker Vertex 70 Fourier transform infrared spectrometer. During the sample preparation, 10 mg grinded carbon foam was homogenized with 250 mg KBr and it was pelletized at 10 tons of load. To identify the crystallinity of the foam and the phases of palladium, X-ray diffraction (XRD) was used. Rietveld analysis was applied to identify and quantitatively characterize the different oxide phases. During the measurements, a Bruker D8 Advance diffractometer (Cu-Kα source, 40 kV and 40 mA) in parallel beam geometry (Göbel mirror) with a Vantec detector was applied. The average crystallite size of the domains was calculated by the mean column length calibrated method by using the full width at half maximum (FWHM) and the width of the Lorentzian component of the fitted profiles. The palladium content of the catalyst was measured by a Varian 720 ES inductively coupled optical emission spectrometer (ICP-OES). For the ICP-OES measurements, the samples were dissolved in nitric acid. The specific surface area (SSA) analysis was carried out by using the nitrogen adsorption-desorption method at 77 K temperature. Micromeritics ASAP 2020 equipment was used, and the evaluation was carried out based on the Brunauer-Emmett-Teller (BET) method. The incorporated nitrogen and the oxygen-containing functional groups were identified by using X-ray photoelectron spectroscopy (XPS). During the measurements, a Kratos XSAM-800 XPS instrument was used with a MgKα X-ray source operated at 120 W (12 kV, 10 mA). Samples were carefully mounted on a double-sided carbon tape, by paying attention to the consistent coverage of the holder. Survey spectra were collected with a pass energy of 80 eV and 1 eV step size. High resolution spectra (C 1s, N 1s, O 1s) were collected with a pass energy of 40 eV and a 0.1 eV step size.

### 2.3. Preparation of the Palladium Decorated Carbon Catalyst

4-Nitroaniline (1.50 g) in powder form and 1 mL concentrated (95 wt%) sulfuric acid were mixed in a ceramic crucible. The mixture was heated by using a Bunsen burner to carbonize the 4-nitroaniline. The formed carbon foams were washed in distilled water five times and the purified carbon sample was dried at 378 K overnight. The activation treatment was carried out in two steps: first, the sample was heated at 673 K for 30 min under nitrogen flow, then it was heated at 1173 K for another 30 min in carbon dioxide atmosphere.

The activated sample (2.00 g) was dispersed in 50 mL distilled water and palladium nitrate solution (0.25 g/20 mL distilled water) was added. The water was evaporated by a rotary vacuum evaporator, and the sample was dried at 378 K overnight. The palladium nitrate impregnated sample was treated at 673 K for 30 min in nitrogen flow, then it was hydrogenated at 673 K for 30 min in hydrogen atmosphere.

### 2.4. Catalytic Tests of the Prepared Carbon Foil Supported Palladium Catalyst

The hydrogenation of nitrobenzene (in methanol) was used as a test system to study the catalytic activity of the final palladium nanocomposite. The concentration of nitrobenzene was 0.25 mol·L^−1^, while 0.1 g of catalyst was added to the system. The reaction was carried out in a Büchi Uster Picoclave reactor which has a 200 mL stainless steel vessel with a heating jacket. The pressure of H_2_ was kept at 20 bar, and the reactants were thermostat at 283 K, 293 K, 303 K, and 323 K. Sampling was carried out after 5, 10, 15, 20, 30, 40, 60, 80, 120, 180, and 240 min. The concentration of nitrobenzene was 0.25 mol·L^−1^ in methanol, and 150 mL of solution and 10, 25, 50, and 75 mg of catalyst were applied during the tests. The efficiency of the catalyst was characterized by calculating the conversion (*X*%) of nitrobenzene based on the following equation (Equation (1)):

(1) $$X\%\;=\;\frac{\;n_{consumed\;NB}}{n_{initial\;NB}}\;\cdot \;100$$

Aniline (AN) yield (*Y*%) was also calculated as follows (Equation (2)):

(2) $$Y\%\;=\;\frac{n{\;}_{formed\;AN\;}}{n_{\;theoritical\;AN\;}}\;\cdot \;100$$

Furthermore, AN selectivity (*S*%) was calculated according to the following equation (Equation (3)):

(3) $$S\%\;=\;\frac{n{\;}_{formed\;AN}}{\sum n_{\;products\;}}\;\cdot \;100$$

## 3. Results and Discussion

### 3.1. Characterization of the Prepared Carbon Foam Support and the Palladium Catalyst

**Figure 1:**
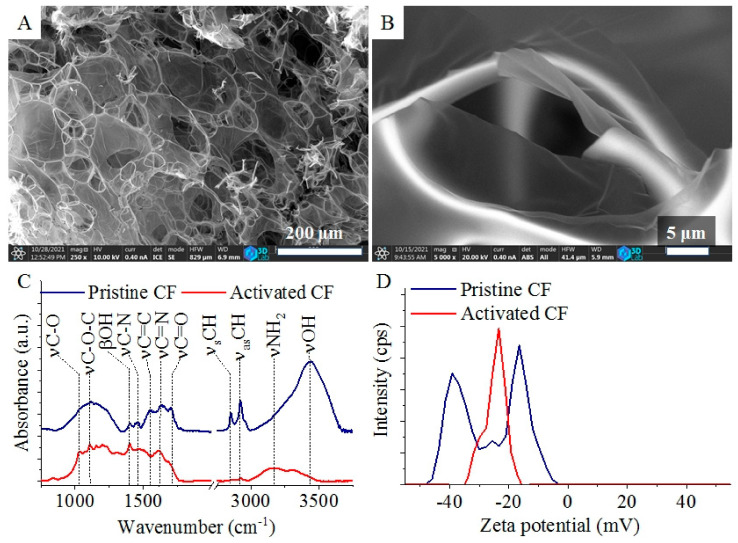
SEM images of the carbon foam (**A**) and its carbon foil (**B**). FTIR spectra (**C**) and zeta potential distributions (**D**) of pristine and activated carbon foils (CFs).

The carbon foam was examined by using SEM, which verified the foam structure of the carbon and the presence of membranous carbon foils (CF) (Figure 1A,B). The carbon foils are stretched out like windows between the walls of the foam cells, and these nitrogen-doped carbon foils were used as supports for palladium nanoparticles during the catalyst preparation. The specific surface is one of the most important properties of the support as it can influence the adsorption capacity of the catalyst. Therefore, the carbon foils’ surface area was determined based on CO_2_ adsorption measurements, by using the Dubinin-Radushkevich isotherm. The surface area of the pristine carbon foils was 182.2 m^2^/g, which increased to 511.7 m^2^/g after activation at 1173 K in CO_2_ atmosphere.

The carbon foils were further examined by using FTIR before (pristine CF) and after activation (activated CF) (Figure 1C). On the FTIR spectrum of the pristine CF, oxygen-containing functional groups were identified, which were formed due to the presence of sulfuric acid and the thermal decomposition processes. At 1029 cm^−1^, 1106 cm^−1^, 1701 cm^−1^, and 3432 cm^−1^, four bands were located which belong to the stretching vibration modes of the C-O, C-O-C, C=O, and -OH groups, and the latter may indicate the presence of adsorbed water [44]. On the wide hydroxyl band, there is a shoulder around 3300 cm^−1^ which indicates the presence of other species such as esters or alcoholic groups. After the activation, the hydroxyl band disappeared by the elimination of water (Figure 1C). Another hydroxyl group-related absorption band was located at 1401 cm^−1^. Due to the incorporated structural nitrogen, the C-N, C=N, and -NH_2_ groups are also present as the bands indicated at 1458 cm^−1^, 1625 cm^−1^, and 3167 cm^−1^ wavenumbers. The band at 1561 cm^−1^ can be associated with the skeleton vibration mode of the C=C bonds, while the symmetric and asymmetric vibration of the -CH bonds are visible at 2855 cm^−1^ and 2929 cm^−1^. The intensity of various bands (e.g., νCH, νC-N) changed after the activation of the carbon foils. This can be explained by the elimination and decomposition of polyaromatic or organic compounds which were formed during the synthesis of the carbon foils. The oxygen and the nitrogen atoms of the -OH and -NH_2_ functional groups become electron rich after deprotonation in the aqueous phase. This led to an increased negative surface charge of CFs, which is indicated by the negative electrokinetic (zeta) potential, measured in the aqueous phase.

Multimodal distribution of the zeta potential values was observed (Figure 1D). In the case of the non-activated pristine CF sample, three peaks were found at −16.1 ± 3.96 mV, −26.6 ± 2.48 mV, and −37.4 ± 3.81 mV electrokinetic potential values. After the activation step, the zeta potential distribution changed and only one peak is visible at −24.8 ± 3.99 mV. The change of the zeta potential can be explained by the elimination of the polyaromatic compounds and amorphous carbon forms next to the graphitized structures by the activation step (at 673 K in N_2_ and at 1173 K in CO_2_). Due to the negative zeta potential, the activated carbon foils are easily dispersed in the aqueous phase or solutions of the catalytically active noble metal (e.g., Pd) salts. The high dispersibility of the CFs contribute to the homogenous adsorption of the palladium(II) ions on their surface. The presence of dissociable nitrogen- and oxygen-containing functional groups on the surface of the CFs initiate the anchoring of the catalytically active metal ions through ion-exchange adsorption and electric interactions.

**Figure 2:**
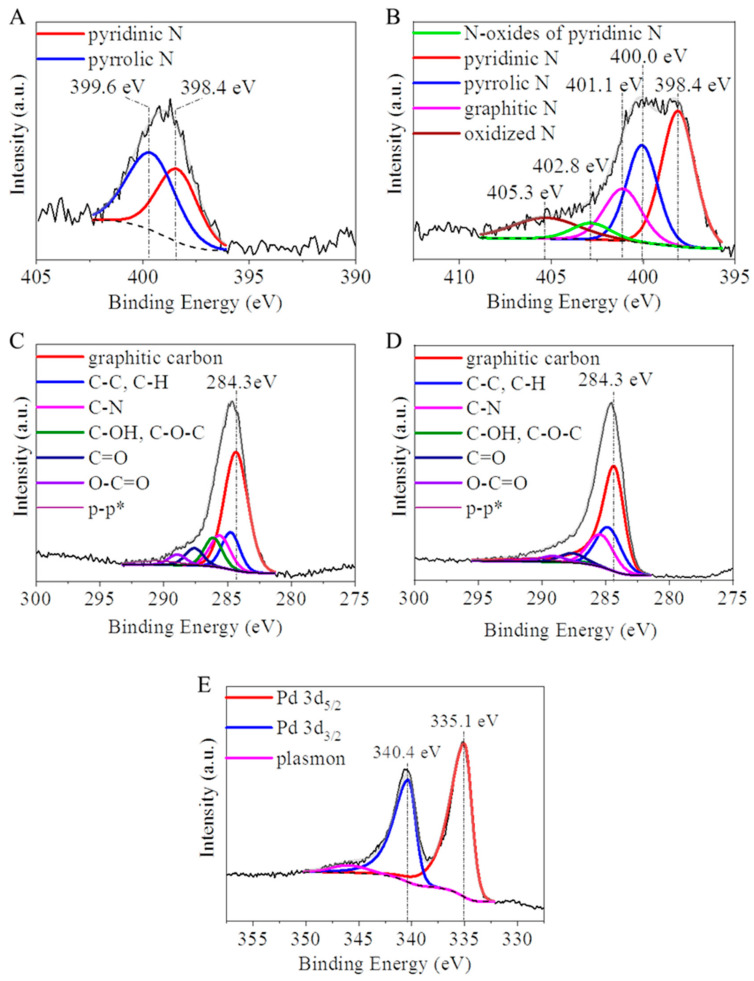
XPS spectra of the deconvoluted N 1s band of the non-activated carbon foil (CF) (**A**) and the Pd/CF catalyst (**B**) sample. The deconvoluted C 1s band of the non-activated CF (**C**) and the Pd/CF catalyst (**D**). Deconvoluted Pd 3d band of the Pd/CF catalyst (**E**).

In the structure of the carbon foils, N atom incorporation usually occurs in three different ways, as pyridinic, pyrrolic, and quaternary nitrogen. These different nitrogen forms contribute in varying degrees to the electron distribution of the nanomaterial, which affects its adsorption and catalytic behavior. In the case of the pristine, non-activated CF sample, on the XPS spectrum of the deconvoluted N 1s band, the presence of pyridinic and pyrrolic nitrogen types were verified as bands were located at 399.6 eV and 398.4 eV binding energies (Figure 2A). Based on the XPS results, the atomic percent of the pyridinic nitrogen was 44.4 at.%, while the pyrrolic was 55.6 at.% in the pristine CF. The carbon foil sample was activated in CO_2_ (at 1173 K) and used for palladium catalyst preparation. The oxidative treatments after the hydrogenation of the catalyst led to the oxidation of the nitrogen forms in the structure. These changes are well observable with the change of the N 1s band, whereas new bands at 402.8 eV and 405.3 eV were identified, which belong to the pyridinic N-oxide and oxidized nitrogen types (Figure 2B). After the activation and reductive heat treatment, a graphitic nitrogen band at 401.1 eV also appeared.

After the deconvolution of the C 1s band, different oxygen-containing bonds are identifiable (Figure 2C,D), which are also visible on the FTIR spectra (Figure 1C). The graphitic carbon was identified at 284.3 eV binding energy, which builds up the structure of the carbon foils. The bands at 285.6 eV, 286.1 eV, 287.6 eV, and 288.9 eV belong to the C-N (pyrrolic and pyridinic nitrogen), C-O (alcohol and ether), C=O (carbonyl), and O-C=O (ester) groups, respectively (Figure 2C,D). The peak at 286.1 eV almost disappeared after the activation (Figure 2D), which can be explained by the hydrogenation of the alcoholic hydroxyl groups.

The presence of elemental palladium in the synthesized Pd/CF catalyst samples was verified by one pair of bands at 340.4 eV and 335.1 eV, respectively (Figure 2E). Other bands which could indicate the presence of palladium oxides are not visible.

The real palladium content of the CF supported catalyst was measured by ICP elemental analysis, and it was found as high as 3.79 wt%.

**Figure 3:**
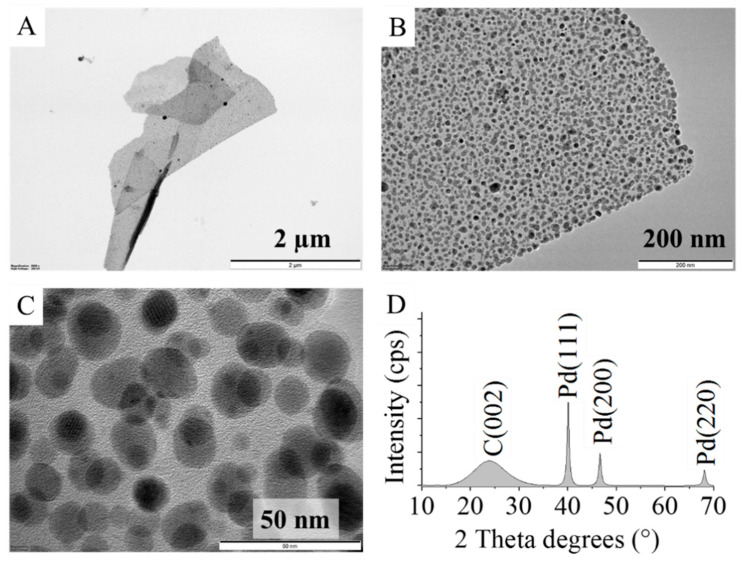
TEM images of the palladium decorated carbon foil (**A**,**B**) and the Pd nanoparticles on the surface (**C**). XRD pattern of the Pd decorated carbon foil catalyst (**D**).

In the TEM images of the Pd decorated carbon foils, membranous carbon structures were visible, which were homogeneously and richly covered by palladium nanoparticles (Figure 3A–C). The Pd particles showed a high degree of dispersity, because 90% of them were between 2.0 and 10.0 nm, while 5% of them were between 10.0 and 19.0 nm, and the average particle size was 5.2 ± 3.7 nm (Supplementary Figure S1). However, based on the XRD measurements the average size was found to be 12 ± 3 nm, which is different from the TEM results.

On the XRD pattern, reflexions at 40.1°, 46.6°, and 68.1° two-theta degrees were identified, which belong to the Pd(111), Pd(200), and Pd(220) phases (Figure 3C). The C(002) reflexion is located at 24.1° two-theta degrees. Based on the XRD results, the reduction step of the Pd impregnated carbon foils in hydrogen atmosphere was successful, because the catalyst contains only a metallic palladium phase, which was also verified by the XPS measurements.

**Figure 4:**
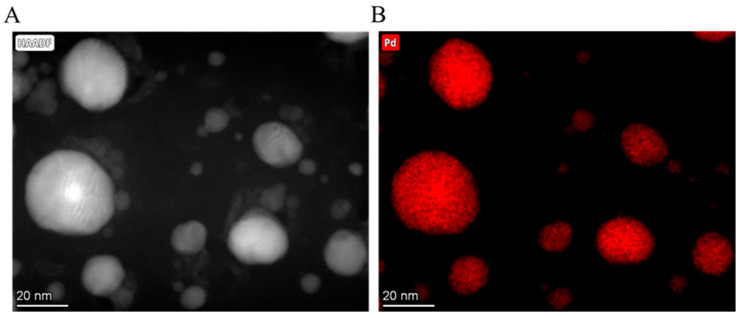
High-angle annular dark field (HAADF) image (**A**) and elemental mapping of the palladium nanoparticles on the surface of the carbon foil (**B**).

In order to clarify and confirm the location of the Pd particles on the surface of the carbon foil support, elemental mapping analysis was carried. On the high-angle annular dark field (HAADF) image, nanoparticles are visible with a strong contrast, and on the elemental mapping these particles were identified as palladium (Figure 4A,B).

The HRTEM images do not show difference between the palladium free and palladium loaded carbon foil (Figure 3 and Supplementary Figure S2). The carbon foil retains its structure even after palladium deposition, and thus it is stable, which is an important requirement for catalyst supports.

### 3.2. Catalytic Tests of the Prepared Palladium Decorated Carbon Foils (Pd/CFs)

**Figure 5:**
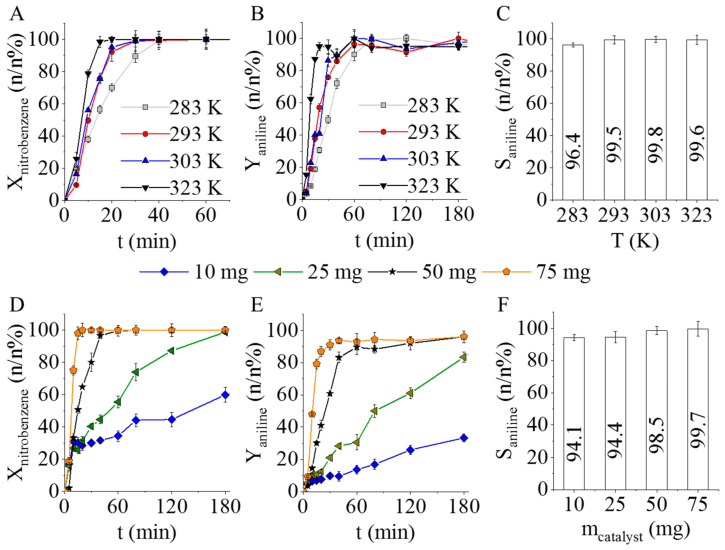
Nitrobenzene conversion (**A**) and aniline yield (**B**) vs. hydrogenation time, and aniline selectivity at four different temperatures after 60 min hydrogenation (**C**) by using 100 mg of Pd/CF catalyst. Nitrobenzene conversion (**D**) and aniline yield vs. hydrogenation time (**E**), and aniline selectivity (**F**) by using of 10 mg, 25 mg, 50 mg, and 75 mg catalyst after 60 min hydrogenation.

The Pd/CF catalyst was tested in nitrobenzene hydrogenation at four different temperatures and 20 bar hydrogen pressure (Figure 5A–C). The concentration of the reactants, by-products, and intermediates were measured by using an Agilent 7890A gas chromatograph, which was coupled with an Agilent 5975C Mass Selective detector.

After 60 min of hydrogenation, the total amount of nitrobenzene was converted to aniline, and the conversion was almost 100%. By increasing the reaction temperature, the elimination of nitrobenzene became faster (Figure 5A). After 1 h, the measured aniline yields were higher than 96.4 n/n%, 99.1 n/n%, and 99.2 n/n% at 293 K, 303 K, and 323 K, respectively. At 283 K, the aniline yield was slightly lower, 90.1 n/n% (Figure 5B). The selectivity was also calculated after 60 min in each case, and it was >99 n/n% at 293 K, 303 K, and 323 K, while at 283 K it was a bit lower (96.4 n/n%). All in all, by applying the prepared palladium decorated carbon foils, outstanding selectivity was achieved regardless of the reaction temperature (Figure 5C).

In each of these experiments, 100 mg of catalyst was used. At 323 K, after 20 min of hydrogenation, the total amount of nitrobenzene was eliminated. Therefore, it was examined whether a smaller amount of catalyst can also provide similar nitrobenzene conversion. Four different amounts of catalyst (10, 25, 50, and 75 mg) were used during the tests (Figure 5D–F). It was found that at least 75 mg of catalyst has to be applied to reach total nitrobenzene conversion within 20 min of hydrogenation (Figure 5D). Since the same conversion and selectivity values can be achieved by using even less catalyst, 50 mg was found to be the optimal quantity. Three parallel measurements were performed for each experiment, and the RSD% was below 10% in most cases with the exception of a few scatter points, which is acceptable for similar systems. Thus, the reproducibility of the measurements is appropriate.

The efficiency of the catalyst was compared to other catalytic systems in nitrobenzene hydrogenation by using the MIRA21 database [45]. The database contains 143 different catalysts tested in NB hydrogenation and provides a score and ranking for each catalyst based on the metal content, nitrobenzene conversion, converted number of moles, aniline yield, and necessary reaction parameters. The prepared Pd/CF has a MIRA21 score of 11.6, with which it ranks as the 11th best catalyst within the database, and thus it is a part of the top 10%.

**Figure 6:**
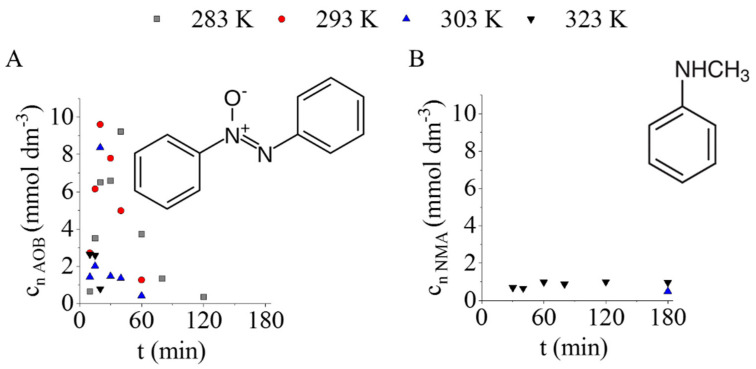
The concentration of azoxybenzene (intermediate) (**A**) and *N*-methylaniline by-product (**B**) vs. time of hydrogenation at various temperatures.

Due to the high catalytic activity of the prepared Pd/CF system, the transformation of NB to AN was very fast; thus, only one intermediate, azoxybenzene (AOB), was detected besides the main product (Figure 6A). During the reaction, the total amount of AOB was converted to aniline. Only one other molecule, *N*-methylaniline (NMA), was identified as a by-product in negligible quantities (less than 1 mmol dm^−3^), and it was formed only at elevated reaction temperatures (>300 K) (Figure 6B).

**Figure 7:**
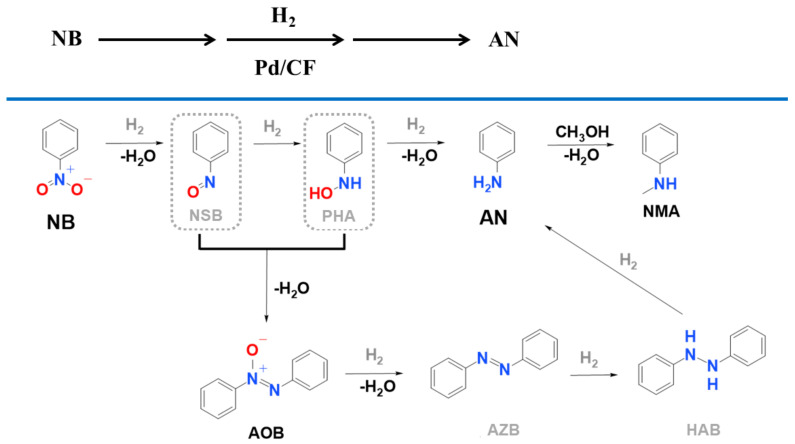
Proposed mechanism of nitrobenzene (NB) hydrogenation to produce aniline (AN) by using the developed palladium decorated carbon foil (Pd/CF) catalyst. The formation of azoxybenzene (AOB, intermediate) and *N*-methylaniline (NMA, by-product) is also shown along with undetected possible intermediates NSB (nitrosobenzene), PHA (*N*-phenylhydroxylamine), AZB (azobenzene), and HAB (hydrazobenzene).

NMA formation can be explained by the methylation of aniline in the presence of methanol, which was used as a solvent during the reactions (Figure 7).

At 323 K, the formation of NMA was started after 30 min of hydrogenation, and by this time the nitrobenzene had completely disappeared, and the aniline yield was above 94 n/n%. A similar tendency can be observed at 303 K, because the appearance of NMA occurred only after 180 min of hydrogenation, and the aniline yield was 98 n/n%. Thus, the methylation of aniline can be avoided by finetuning the reaction conditions and choosing the optimal reaction time and temperature.

**Figure 8:**
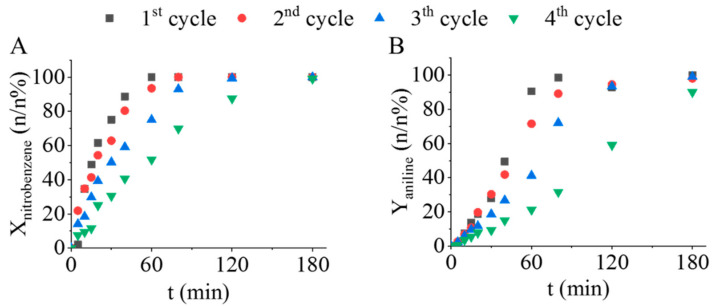
Nitrobenzene conversion (**A**) and aniline yield (**B**) vs. time of hydrogenation during the reuse tests of the prepared Pd/CF catalyst.

Using catalysts effectively more than once is an important feature; thus, the prepared Pd/CF sample was examined, and reuse tests were carried out in four cycles without regeneration (Figure 8A,B).

The reuse tests were carried out at 303 K, because according to the previous catalytic tests NMA was not formed at this temperature and only 50 mg of catalyst was used. For two cycles, a significant decrease in the catalytic activity was not observed, and total nitrobenzene conversion was reached after 80 min, while aniline selectivity was >92 n/n% after 120 min of hydrogenation. However, a significant decrease was observed in the 4th reuse test, and the reaction became slower, but the nitrobenzene conversion was still 99.1 n/n% and the maximum aniline yield was 90.0 n/n% after three hours of hydrogenation. *N*-methylaniline was not detectable during the reuse tests. The total amount of azoxybenzene (intermediate) was converted to aniline by the end of the hydrogenation in all experiments. The aniline selectivity was above 99 n/n% during the first three cycles, while after the 4th cycle, a slight decrease was experienced, and the maximum selectivity was 97.9 n/n%. ICP-OES measurement was carried out on the used catalyst after the recycling test (after four cycles). The measured palladium content was 3.07 wt%, which decreased by 19% compared to the initial Pd loading (3.79 wt%) and led to a slightly lower catalytic activity. The prepared Pd/CF catalyst can be successfully applied in nitrobenzene hydrogenation in three catalytic cycles without regeneration, but after that, the regeneration of the system is necessary.

## 4. Conclusions

Nitrogen-doped carbon foam was successfully prepared within which carbon foils were stretched out like windows between the walls of the foam cells. The activated carbon foils incorporated oxygen-containing functional groups (-COOH, -OH), which can be deprotonated in the aqueous phase; thus, their polar feature leads to excellent wettability and dispersibility in water. Due to the presence of these groups on the surface of the foils, the electrokinetic (zeta) potential of the system was sufficiently negative (−24.8 ± 3.99 mV and −37.1 ± 1.04 mV) to reach electrostatic stabilization. These nitrogen-doped carbon foils were decorated by palladium nanoparticles through electrostatic- and ion-exchange adsorption. The carbon surface was richly covered by palladium nanoparticles and the location of those on the surface was homogenous. The Pd decorated carbon foils were tested in nitrobenzene hydrogenation. The prepared Pd/CF system showed high catalytic activity in the nitrobenzene conversion to aniline. Total conversion was achieved after 60 min, along with 99.1 n/n% aniline yield by using 100 mg of catalyst at 303 K. The catalyst is highly selective towards aniline because by-products were not found at 303 K after 1 h of hydrogenation, and the aniline selectivity was 99.8 n/n%. All in all, a highly efficient carbon-based catalyst support was prepared by the carbonization of 4-nitroaniline. The preparation was very easy and fast, and the support was efficiently decorated by palladium nanoparticles. The final Pd/CF catalyst has excellent properties and can be successfully applied in the hydrogenation of nitrobenzene. All in all, an easy to prepare catalyst was developed successfully with high catalytic activity and excellent aniline selectivity.

## Data Availability

Additional figures are available in the Supplementary Materials.

## Supplementary Materials

The following supporting information can be downloaded at: https://www.mdpi.com/article/10.3390/ijms23126423/s1.
