# Supplementary material for: Palladium Decorated N-Doped Carbon Foam as a Highly Active and Selective Catalyst for Nitrobenzene Hydrogenation
Source: Int J Mol Sci. 2022 Jun 8;23(12):6423. doi: 10.3390/ijms23126423 (PMC9223379; doi:10.3390/ijms23126423)
Supplement: Supplementary file 1 [file ijms-23-06423-s001.zip › ijms-1751561-supplementary.pdf]

Supplementary Information

Palladium Decorated N-doped Carbon Foam as Highly Active and Selective Catalyst for Nitrobenzene Hydrogenation

Ádám Prekob[1,a], Ákos Szamosvölgyi[2,b], Gábor Muránszky[1,c], János Lakatos[1,d], Zoltán Kónya[2,e], Béla Fiser[3,4,f], Béla Viskolcz[1,g] and László Vanyorek[1,h,*]

[1]Institute of Chemistry, University of Miskolc, 3515 Miskolc-Egyetemváros, Hungary
[2]University of Szeged, Department of Applied and Environmental Chemistry,6720 Szeged, Rerrich Béla tér 1. Hungary
[3]Higher Education and Industrial Cooperation Centre, University of Miskolc, 3515 Miskolc-Egyetemváros, Hungary
[4]Ferenc Rákóczi II. Transcarpathian Hungarian College of Higher Education, 90200 Beregszász, Transcarpathia, Ukraine
[a]kempadam@uni-miskolc.hu, [b]akos.szamosvolgyi@gmail.com, [c]kemmug@uni-miskolc.hu, [d]mtasotak@uni-miskolc.hu, [e]konya@chem.u-szeged.hu, [*,f]kemfiser@ uni-miskolc.hu, [g]bela.viskolcz@uni-miskolc.hu, [*,h]kemvanyi@uni-miskolc.hu.

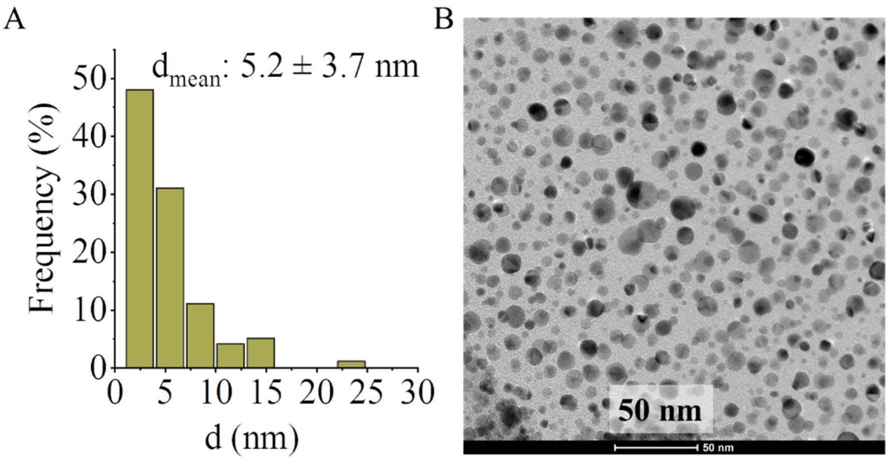

C

Mean	$S_{D.}$	Min.	Max.	Median	P90	P95
5.2 nm	3.7 nm	2.0 nm	22.6 nm	4.2 nm	10.0 nm	18.9 nm

Figure S1. Size distribution (A), HRTEM image (B), and size distribution data (C) of the prepared palladium nanoparticles.

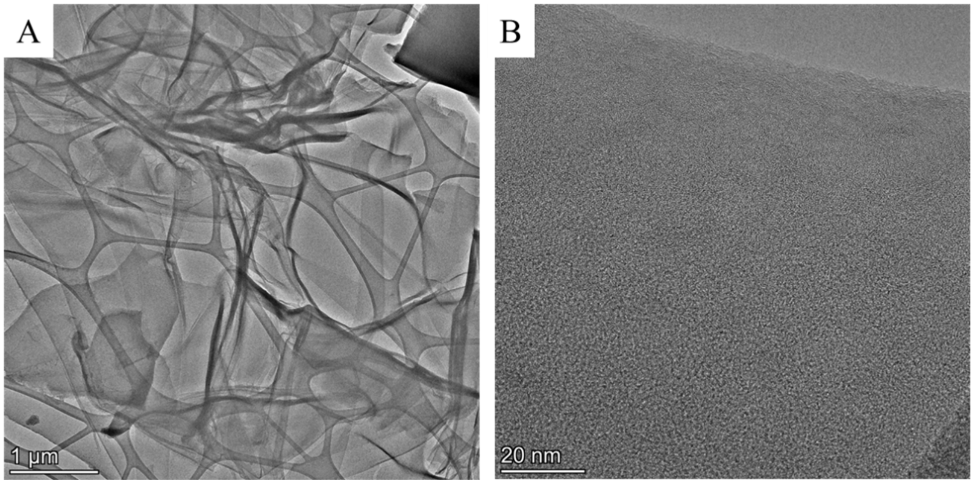

Figure S2. HRTEM images of the prepared carbon foils at different resolutions: 11 000x (A) and 500 000x (B)
